# Breast cancer survival after mammography dissemination in Brazil: a population-based analysis of 2,715 cases

**DOI:** 10.1186/s12905-023-02803-4

**Published:** 2023-12-04

**Authors:** Juliana O. Fernandes, Beatriz F. Machado, Cassio Cardoso-Filho, Juliana Nativio, Cesar Cabello, Diama B. Vale

**Affiliations:** 1 Department of Obstetrics and Gynecology, University of Campinas, Women’s Hospital, Unicamp. Rua Alexander Fleming, 101, 13083-881, Cidade Universitária, Campinas, SP Brazil; 2 Surveillance Section, Municipal Health Department, Campinas City Hall, Paço Municipal, Avenida Anchieta, nº 200, 13.015-904, Campinas, SP Brazil

**Keywords:** Breast neoplasms, Survival analysis, Neoplasm staging, Early detection of cancer, Mortality

## Abstract

**Background:**

This study aims to assess breast cancer survival rates after one decade of mammography in a large urban area of Brazil.

**Methods:**

It is a population-based retrospective cohort of women with breast cancer in Campinas, São Paulo, from 2010 to 2014. Age, vital status and stage were accessed through the cancer and mortality registry, and patients records. Statistics used Kaplan–Meier, log-rank and Cox's regression.

**Results:**

Out of the 2,715 cases, 665 deaths (24.5%) were confirmed until early 2020. The mean age at diagnosis was 58.6 years. Women 50–69 years were 48.0%, and stage I the most frequent (25.0%). The overall mean survival was 8.4 years (8.2–8.5). The 5-year survival (5yOS) for overall, 40–49, 50–59, 60–69, 70–79 years was respectively 80.5%, 87.7%, 83.7%, 83.8% and 75.5%. The 5yOS for stages 0, I, II, III and IV was 95.2%, 92.6%, 89.4%, 71.1% and 47.1%. There was no significant difference in survival in stage I or II (*p* = 0.058). Compared to women 50–59 years, death's risk was 2.3 times higher for women 70–79 years and 26% lower for women 40–49 years. Concerning stage I, the risk of death was 1.5, 4.1 and 8.6 times higher, and 34% lower, respectively, for stage II, III, IV and 0*.*

**Conclusions:**

In Brazil, breast cancers are currently diagnosed in the early stages, although advanced cases persist. Survival rates may reflect improvements in screening, early detection and treatment. The results can reflect the current status of other regions or countries with similar health care conditions.

## Background

Breast cancer is a significant public health problem, being one of the leading causes of mortality and morbidity that affects the female population between 40 and 69 years old [1]. When diagnosed at early stages, it has great chances of cure. In selected countries, survival rates can reach 90% in five years [2]. Since the number of breast cancer survivors has increased, survival analysis describes disease behaviour and related prognostic factors.

The elements that stand out the most in the prognosis of women with breast cancer are age at diagnosis, size of the tumour, staging, therapeutic management, delays in treatment, race and socioeconomic status [3]. At the early stages, treatments are less aggressive, and morbidities lower, resulting in higher survivals [4]. The five-year survival rates in stages I and II (early breast cancer – E.B.C.) range from 80 to 99%, while in a more advanced stage with metastasis (stage IV), the survival rate may drop to less than 30% [5]. Barriers to accessing screening are challenges women face in low- and middle-income countries to have an early diagnosis. Limited access to therapeutic modalities may also lead to reduced survivals, regardless of the stage [2, 6].

Breast cancer is most frequently diagnosed in women in the post-menopausal period, between 55 and 64 years old [5]. About 20% occur in women below this age-group. These cases tend to present more aggressive tumours, with a greater chance of developing resistance to treatment, therefore lowering survival rates [7, 8].

Survival rates vary somewhat around the world. In the U.S.A., the five-year survival rate is 90% (2010–2016), and in England 85% (2013–2017) [5, 9]. In Latin American and Central American countries in general, this rate is around 80% [2]. Brazil's last population-based survival rate reported figures of 74.3% from 2000 to 2014 [10]. The scarcity of quality data in low and middle-income countries makes it challenging to implement public cancer control policies.

Adequate data to calculate cancer incidence and survival rates come from population-based cancer registries (P.B.C.R.). Campinas has a P.B.C.R. integrating the database of the National Cancer Institute [11]. It is an urban city with 1.2 million inhabitants, presenting social inequalities similar to those observed in other large cities of middle-income countries [12]. According to age and stage at diagnosis, this study aims to assess the overall survival of breast cancer rates in Campinas, based on data from the P.B.C.R. from 2010 to 2014. This period reflects one decade of mammography spread in the region for screening and early detection of cancer. From 2000 to 2012, an increase of threefold in the number of mammograms performed was observed [13]. The results of this study can guide policymakers to improve health care for this and other similar areas.

## Methods

It was a retrospective cohort study of women diagnosed with breast cancer in Campinas between 2010 and 2014. The P.B.C.R. includes all cancer cases of women living in the city, regardless of the type of health assistance provided (public or private). After excluding duplicates and cases registered by death certificates, 2,715 cases were last in the database. The data from the P.B.C.R. and the Mortality Information System (M.I.S.) records were the sources of primary data. When data were lack, patient's records from the municipality's health services were accessed. Women were diagnosed by public or private services in the city. Only Campinas residents are included in the P.B.C.R.

To be defined as 'deaths', cases should be registered as such in the databases. The remaining cases were considered 'alive'. Of those alive, 15% were accessed through records on the region's hospitals or clinics and censored on the last registered follow-up date. The remaining were considered alive until March 31, 2020, when the COVID-19 pandemic spread in Brazil and the isolation period started. Around this date, 37,5% of these women had an active financial transaction record (Individual Taxpayer Register – C.P.F., available for public consultation).

The following variables were available and included for analysis: age at the date of diagnosis, vital status, date of death, date of censorship/last follow-up, and stage at diagnosis—according to the American Joint Committee on Cancer [14]. The molecular profile was unavailable during the study period. Crossing information through systems and the different records accessed enable to correct misrecording data at the P.B.C.R.

Overall survival was calculated by the time from the date of diagnosis (histopathologic result) until the date of death or until the study's censorship or end date. Statistics was performed by the Kaplan–Meier method and expressed as percentages. The log-rank test compared the different survival curves of the different categories of variables. Cox's regression models were applied to assess factors related to survival and to estimate risk ratios. For statistical analysis, the computer program S.A.S. for Windows (Statistical Analysis System), version 9.2, was employed. (S.A.S. Institute Inc, 2002–2008, Cary, NC, U.S.A.).

This study is part of a regular research project funded by F.A.P.E.S.P. under number 2017/21908–1, and was approved by the Unicamp Research and Ethics Committee under number CAAE 89399018.2.0000.5404. The funder had no involvement in the study apart of supporting financially. The Committee waived the need for Informed Consent. The confidentiality was guaranteed, and personal data was handled only by the P.B.C.R. and Mortality Surveillance Department teams as part of their routine activities. Two researchers from the study team (J.F.O., B.F.M.) had actively collected data in hospitals and clinics, completing data on stage and follow-up missing in the P.B.C.R..

## Results

**Fig. 1:**
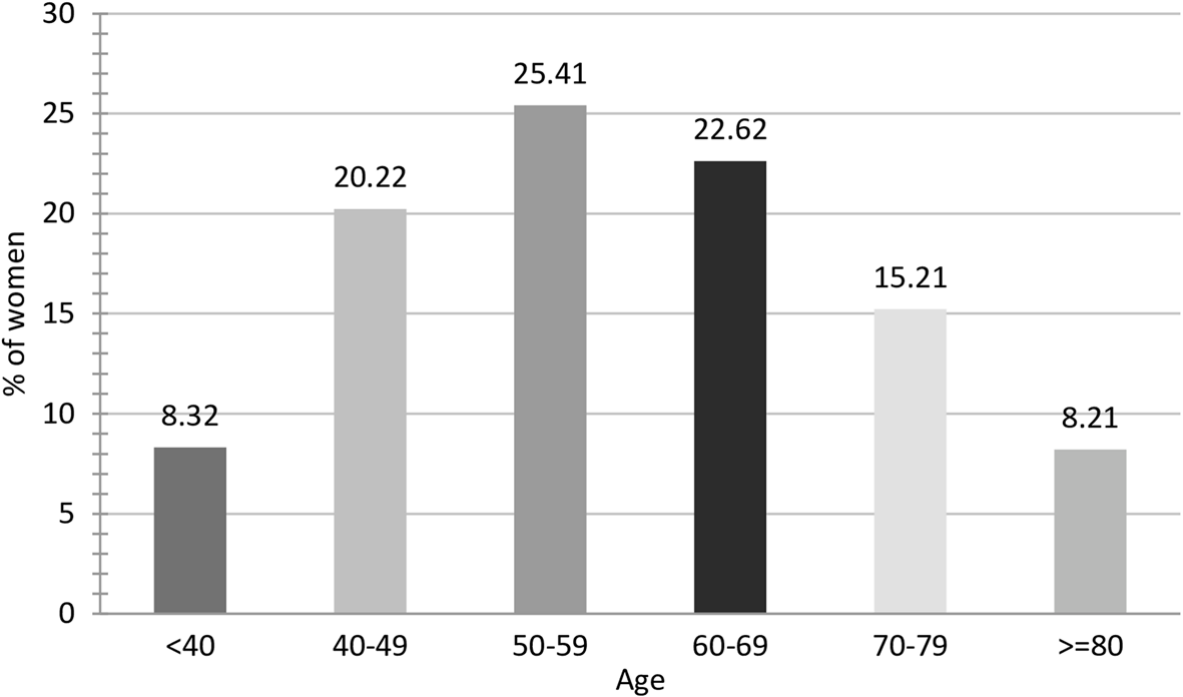
Distribution of women with breast cancer according to the age group at diagnosis (*n* = 2,715)

Of the 2,715 cases recorded between 2010 and 2014, 665 deaths (24.5%) were confirmed until March 31, 2020. From the total sample, in 2,054 women (75.7%), it was possible to determine the stage at diagnosis: 1,547 in the group of alive women (75.5%) and 507 in the group of dead women (76.2%) (*p* = 0.685). The mean age at diagnosis was 58.6 years (median 58.0, standard deviation—SD 12.97), being 56.9 years among the alive and 64.0 years among the dead (*P* < 0.001). Women between 50 and 69 represented 48.0% of the sample, and those under 50 28.5% (Fig. 1).

**Fig. 2:**
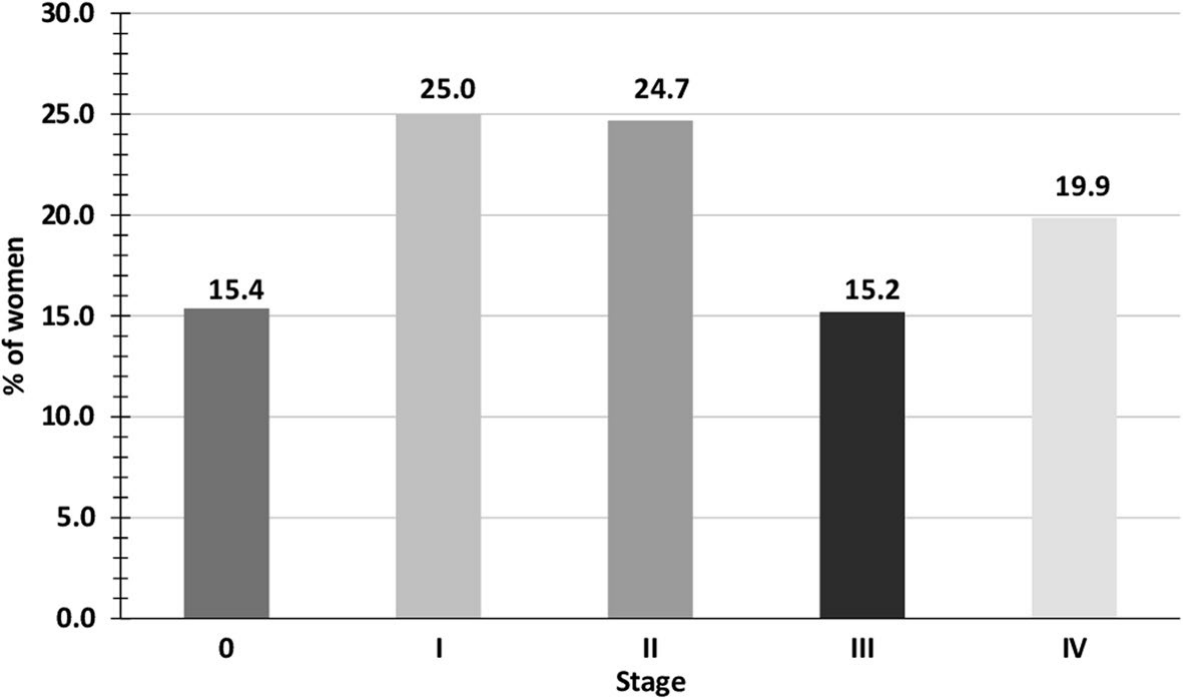
Distribution of women with breast cancer according to the stage at diagnosis (*n* = 2,054)

In women in whom it was possible to determine the stage at diagnosis, stage I was the most frequent (25.0%) (Fig. 2). Stage 0 (in situ) corresponded to 15.4% and stage IV to 19.9%. The comparison between women known and unknown stage showed an average age for those staged of 57.7 years (median 57.0, SD 13.7) and for that not-staged of 61.6 years (median 61.0, SD 15.2) (*p* < 0.001). The median survival of women staged was 6.3 years (median 6.6, SD 2.62) and that of women not-staged of 6.5 years (median 6.9, SD 2.6) (*p* = 0.027) (data not shown). The median survival was undetermined in most age groups and stages.

The overall mean survival (mOS) of the sample was 8.4 years (95% Confidence Interval – CI 8.2–8.5), the overall 5-year survival (5yOS) 80.5% and the overall 10-year survival (10yOS) 69.9%. The highest mOS was in women aged 40 to 49 years (9.1 years; 95% CI 8.9–9.3), and the shortest in the older group—women over 80 years (5.7 years; 95% CI 5.2 -6.2). The 5yOS in women younger than 40 years was 82.2%, significantly lower than for women aged 40 to 49, 87.7% (*p* = 0.002). Up from 50 to 69, 5yOS decreased with increasing age: 83.7% for those aged 50 to 59 years and 83.8% for those aged 60 to 69 years, 75.5% for those aged 70 to 79 and 51.4% for women older than 79 years. Table 1 shows the mOS, 5yOS and 10yOS for the different age groups.

**Table 1:** Overall survival of 2,715 women with breast cancer according to the age-group at diagnosis

Age (years)	Mean^a^ Overall Survival (95% CI)	Five-years Overall Survival (SD)	Ten-years Overall Survival (SD)
< 40	8.5 years (8.1–8.9)	82.2% (2.5%)	68.7% (5.9%)
40 to 49	9.1 years (8.9–9.3)	87.7% (1.4%)	82.1% (2.4%)
50 to 59	8.7 years (8.5–9.0)	83.7% (1.4%)	78.6% (2.5%)
60 to 69	8.6 years (8.3; 8.9)	83.8% (1.5%)	71.3% (3.6%)
70 to 79	7.6 years (7.3–7.9)	75.5% (2.1%)	55.2% (4.1%)
≥ 80	5.7 years (5.2–6.2)	51.4% (3.4%)	34.0% (4.8%)

*95% CI* 95% Confidence Interval*, SD* Standard Deviation

^a^The median overall survival was over 10 years but undetermined in the age groups < 40, 40 to 49, 50 to 59, 60 to 69, and overall. The median overall survival was 10.1 years (9.1–11.1) in 70 to 79 years and 5.2 years (3.3–7.1) in ≥ 80

**Fig. 3:**
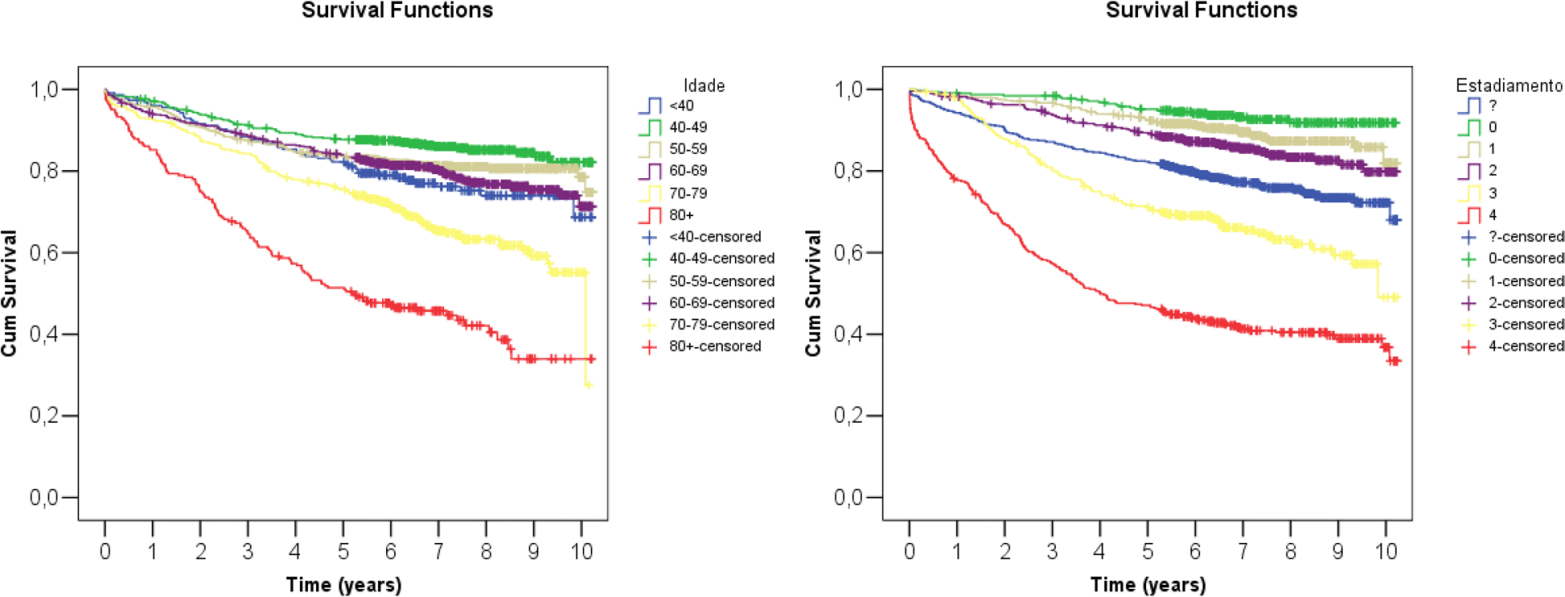
Kaplan-Meyer survival curves for women diagnosed with breast cancer as a function of age and stage (*n* = 2,715)

The 5yOS for stages 0 (in situ), I, II, III and IV was 95.2%, 92.6%, 89.4%, 71.1% and 47.1% (Table 2). Pairwise Comparisons (Log Rank, Mantel-Cox tests) showed a significant difference in survival of stage 0 (in situ) and all others. There was no significant difference in the survival of women diagnosed with early-stage breast cancer (E.B.C., stage I or II) (*P* = 0.058). The Kaplan-Meyer curves are seen in Fig. 3.

**Table 2:** Overall survival of 2,054 women with breast cancer according to the stage at diagnosis

Stage	Mean^a^ Overall Survival (95% CI)	Five-years Overall Survival (SD)	Ten-years Overall Survival (SD)
Stage 0 (in situ)	9.7 years (9.5–9.9)	95.2% (1.2%)	91.8% (1.8%)
Stage I	9.5 years (9.3–9.6)	92.6% (1.2%)	81.9% (4.4%)
Stage II	9.1 years (8.9–9.4)	89.4% (1.4%)	79.8% (2.8%)
Stage III	7.6 years (7.2–8.0)	71.1% (2.6%)	49.0% (8.3%)
Stage IV	5.4 years (5.0–5.8)	47.1% (2.5%)	36.8% (3.3%)

*95% CI* 95% Confidence Interval*, S.D.* Standard Deviation

^a^The median survival was over 10 years but undetermined in stages 0 (in situ), I, II, III, and overall. Stage IV's median survival was 4.0 years (3.0–5.1)

Univariate and multivariate analysis were performed to check the influence of age and stage (Table 3). Compared to women between 50 and 59 years old, the multivariate analysis obtained a risk of death 2.3 times and 4.2 times higher, respectively, for women 70 to 79 years old and women more aged than 79 years. For women 40 to 49 years, the risk was 26% lower. Concerning stage I, the risk of death was 1.5, 4.1, 8.6, 2.1 times higher and 34% lower, respectively, for women with stage II, III, IV, unknown and 0 *(*in situ*).*

**Table 3:** Uni and multivariate COX regression analysis of survival in women with breast cancer (*n* = 2,715)

	**Univariate Analysis**			**Multivariate Analysis**		
**Age (y)**	***P*-value**	**HR**	**95% CI**	***P*-value**	**HR**	**95% CI**
** < 40**	0.111	1.29	0.94–1.78	0.339	1.17	0.85–1.61
**40 to 49**	**0.037**	0.74	0.56–0.98	0.033	0.74	0.56–0.98
**50 to 59**	-	1.00	-	-	1.00	-
**60 to 69**	0.273	1.15	0.90–1.46	0.127	1.21	0.95–1.54
**70 to 79**	** < 0.001**	2.02	1.59–2.56	** < 0.001**	2.27	1.79–2.88
** ≥ 80**	** < 0.001**	4.09	3.20–5.22	** < 0.001**	4.16	3.24–5.33
	**Univariate Analysis**			**Multivariate Analysis**		
**Stage**	***P*-value**	**HR**	**95% CI**	***P*-value**	**HR**	**95% CI**
**Stage 0**	**0.049**	0.61	0.37–0.99	0.101	0.66	0.41–1.08
**Stage I**	**-**	1.00	-	**-**	1.00	-
**Stage II**	0.057	1.39	0.99–1.96	0.016	1.53	1.08–2.15
**Stage III**	** < 0.001**	3.72	2.70–5.12	** < 0.001**	4.05	2.94–5.59
**Stage IV**	** < 0.001**	8.00	5.99–10.68	** < 0.001**	8.57	6.41–11.45
**Unknown**	** < 0.001**	2.29	1.69–3.10	** < 0.001**	2.08	1.54–2.82

*H.R.* Hazard Ratio*, 95% CI* 95% Confidence Interval*. *Variables were selected by Stepwise Criteria

## Discussion

In this population-based study that accessed data from 2,715 women with breast cancer in a large city in Brazil, it was observed that the average age of women at diagnosis was 58.6 years, that the diagnoses were early and that the five years overall survival (5yOS) was 80.5%. Survival was significantly lower in more advanced stages and older women. It is the largest Brazilian cohort of survival assessment of the last ten years, just after the period when mammography has significantly spread in Brazil [13].

The universal health care system in Brazil has expanded significantly over the past decade, increasing the provision of services and cancer care qualification. Particularly in São Paulo State, mammography has been established to screen and early detection of breast cancer [13]. A significant increase in mammography access was observed in the region through national cancer control program incentives [15]. The expansion of the chemotherapy offer and the inclusion of new drugs allowed the transition to less radical treatment modalities, with less morbidity, besides being more effective.

Two results were expected in the implementation of these actions: downstaging and improvements in survival. In this study, we observed that one in every four women with breast cancer in Campinas was diagnosed in clinical stage I. A hospital-based cancer registry study of São Paulo state observed a significant upward trend in the proportion of cases diagnosed in stage I and a significant reduction in stage II cases from 2000 to 2015 [16]. This detection of tumours in earlier stages is firmly attributed to expanding the screening and early detection incentives.

In this study, the 5yOS in stages I and II were 92.6% and 89.4%, respectively (*p* = 0.058), which means that the survival of radiologically or clinically detected cancers were. It is an argument to do not support screening. However, it is possible that the morbidity related to the earlier treatment would result in a better quality of life for the survivors. Another interesting result is that women diagnosed in stage 0 (in situ) corresponded to 15.4% of cases, the upper limit recommended for a screening program [17], indicating overuse and possible overdiagnosis.

The observed 5yOS was 80.5%, and the ten years overall survival (10yOS) 69.9%. A population-based study in Barretos, 350 km far from Campinas, reported a slightly lower 5yOS of 74.3% from 2000 to 2015 (*n* = 2,110 cases) [10]. Considering both cities' health care framework are relatively similar, these results are likely to indicate improved treatment assistance in the region. The large global survival surveillance consortium, the CONCORD study, updated its third version pointing to a 5yOS for Brazil of 73.9–76.5% from 2000 to 2014, a period less sensitive to reflect the recent improvements observed in recent years [2].

This 5yOS of 80.5% is lower than that found in high-income countries but higher than low and middle-income countries. It is challenging to consider the differences in the periods of observation [2, 18]. From the public health perspective, this result indicates that Brazil's path in terms of investments resulted in a positive impact. Still, the sustainability of these actions is necessary to maintain the achievements. Age, stage and treatment are the factors that most influence the woman's prognosis. It should be noted that, even though there is a tendency for downstaging in Brazil, the proportion of cases diagnosed in advanced stages is still high [16].

In the multivariate analysis, compared to women in stage I, the risk of death was 34% lower for women in stage 0 *(*in situ*)*, and 1.5, 4.1, 8.6 and 2.1 times higher, respectively for women in stages II, III, IV and unknown. The 5yOS for stages 0, I, II, III and IV was respectively 95.2%, 92.6%, 89.4%, 71.1% and 47.1. These survivals are close to those observed in other national studies [10, 19, 20] and lower than those found in countries like U.S.A. and England [5, 9].

The highest mean and 5yOS were found in the group of women aged 40 to 49 years (9.1 years; 95% CI 8.9–9.3, 87.7%), reducing significantly after 50 years. The multivariate analysis obtained a risk of death 2.3 and 4.2 times higher, respectively, for women between 70 to 79 years old and older than 79 years old; and 26% lower for those between 40 to 49 years old, compared to women between 50 to 59 years old. These results support the evidence that older women have a worse prognosis regardless of the stage at which they are diagnosed [5, 19]. However, very young women (those under 40 years old) had a 5yOS of 82.2%, significantly less than women aged 40 to 49, 87.7% (*p* = 0.002). Other studies also point to reduced survival in very young women [7, 8, 20], probably due to the diagnosis of more aggressive molecular types of tumours found in this group.

This study is the largest population-based cohort published in Brazil in recent years and has benefited from the multiple data sources for its progress. Its results reflect the reality of Brazil's most populated cities, with different access and quality of care, and can guide public policies for cancer control. The current study's solidness dwells on the number of patients and the reliability of the follow-up. The active search of vital status allowed us to reach higher quality to the data presented.

There are two main limitations. The first is that in 25% of cases the stage was unknown. Secondary analyzes of these cases were carried out, and it was observed that the mean age and mean survival were slightly higher among those not staged (*p* < 0.001 and *P* = 0.027, respectively). It may indicate that if it had any influence on the results, it would be discreet.

The second limitation is that in 85% of the cases considered alive, it was impossible to establish the date of censorship. It may have significantly influenced the results of 10yOS and, less significantly, 5yOS an mOS. However, this is a problem inherent in population-based studies. It must be said that death records in São Paulo state are of high quality and that the analysis of the registers of women's economic activities may have mitigated this bias. We consider that given the lack of population-based studies in low and middle-income countries, the data presented is relevant even with its limitation. Its quality should be carefully evaluated compared with data from countries with well-established registration systems.

## Conclusions

In Brazil breast cancer cases are currently diagnosed in the early stages, although advanced cases persist. Survival rates are lower than those observed in high-income countries but may reflect screening, early detection, and treatment improvements.

## Data Availability

The datasets generated during the current study are available from the corresponding author on reasonable request. The Surveillance Section of the Municipal Health Department, Campinas, is the data manager and has specif restrictions to preserve confidentiality, although aggregate data may be shared upon request.

## Abbreviations

5yOS: Overall 5-year survival
10yOS: Ooverall 10-year survival
CPF: Individual Taxpayer Register
EBC: Early breast cancer (stage I or II)
mOS: Overall mean survival
MIS: Mortality Information System
PBCR: Population-based cancer registries
